# Management of rare diseases of the Head, Neck and Teeth: results of a French population-based prospective 8-year study

**DOI:** 10.1186/s13023-017-0650-0

**Published:** 2017-05-19

**Authors:** Lisa Friedlander, Rémy Choquet, Eva Galliani, Myriam de Chalendar, Claude Messiaen, Amélie Ruel, Marie-Paule Vazquez, Ariane Berdal, Corinne Alberti, Muriel De La Dure Molla

**Affiliations:** 1 Université Paris Diderot – Sorbonne Paris Cité, INSERM, Laboratoire ECEVE UMR1123, Paris, France; 2 Centre de référence des malformations rares de la face et de la cavité buccale, Hôpital Rothschild, AP-HP, Paris, France; 3 Banque Nationale de Données Maladies Rares, Hôpital Necker Enfants Malades, AP-HP, Paris, France; 4 Centre de référence des malformations rares de la face et de la cavité buccale, Hôpital Necker, AP-HP, Paris, France; 5 Université Paris Descartes-Sorbonne Paris Cité, Paris, France; 6 Service de chirurgie maxillo-faciale et de chirurgie plastique, Hôpital Necker, AP–HP, Paris, France; 7 Filière de santé maladies rares TETECOU: malformations rares de la tête, du cou et des dents, Hôpital Necker, Paris, France; 8 Université Paris Diderot – Sorbonne Paris Cité, INSERM, Laboratoire de physiopathologie orale et moléculaire, UMRS 1138, Paris, France; 9 INSERM UMR_S1163 Bases moléculaires et physiopathologiques des ostéochondrodysplasies, Institut Imagine, Necker, Paris, France

**Keywords:** Rare diseases, Registry, Epidemiology, Orofacial diseases, Oral cleft

## Abstract

**Background:**

In the last ten years, national rare disease networks have been established in France, including national centres of expertise and regional ones, with storage of patient data in a bioinformatics tool. The aim was to contribute to the development and evaluation of health strategies to improve the management of patients with rare diseases. The objective of this study has been to provide the first national-level data concerning rare diseases of the head, neck and teeth and to assess the balance between demand and supply of care in France.

**Methods:**

Centres of expertise for rare diseases record a minimum data set on their clinical cases, using a list of rare Head, Neck and Teeth diseases established in 2006. The present analysis focuses on 2008 to 2015 data based on the Orphanet nomenclature. Each rare disease RD “case” was defined by status “affected” and by the degree of diagnostic certainty, encoded as: confirmed, probable or non-classifiable. Analysed parameters, presented with their 95% confidence intervals using a Poisson model, were the following: time and age at diagnosis, proportions of crude and standardized RD prevalence by age, gender and geographical site. The criteria studied were the proportions of patients in Paris Region and the “included cases geography”, in which these proportions were projected onto the other French Regions, adjusting for local populations.

**Results:**

In Paris Region, estimated prevalence of these diseases was 5.58 per 10,000 inhabitants (95% CI 4.3-7.1). At December 31st 2015, 11,342 patients were referenced in total in France, of whom 7294 were in Paris Region. More than 580 individual clinical entities (ORPHA code) were identified with their respective frequencies. Most abnormalities were diagnosed antenatally. Nearly 80% of patients recorded come to Paris hospitals to obtain either diagnosis, care or follow up. We observed that the rarer the disease, the more patients were referred to Paris hospitals.

**Conclusions:**

A health network covering a range of aspects of the rare diseases problematic from diagnostics to research has been developed in France. Despite this, there is still a noticeable imbalance between health care supply and demand in this area.

**Electronic supplementary material:**

The online version of this article (doi:10.1186/s13023-017-0650-0) contains supplementary material, which is available to authorized users.

## Background

To be considered as rare, an individual disease must affect a limited number of people within the population. It is defined, in Europe, by a prevalence lower than 1 per 2000 (European Regulations on Orphan Medicine) [1].

More than 7000 such clinical entities have been described of which between 70 and 80% are genetic [2]. In the Orphanet database [3], rare Head and Neck disorders comprise 2153 diseases [4]. These include rare facial disorders (such as lip and cleft palate, isolated or associated with syndromes like Goldenhar syndrome or van der Woude syndrome), oral and dental disorders (such as isolated oligodontia or associated with syndromes such as ectodermal dysplasia), ORL disorders (such as cervicofacial fistulas associated with BOR syndrome), and cranial disorders (such as craniofaciosynostosis associated for example with Crouzon syndrome).

These rare diseases may be life threatening, cause growth disorders, and impose an economic and social burden because of their repercussions on psychological and physiological growth [5, 6]. Many syndromes present facial anomalies. For some of these anomalies no treatment is required, for example hypertelorism, but for many others surgery is necessary to restore appearance and function [7, 8]. Facial dismorphologies have repercussions for oral functions: breathing, chewing, deglutition, phonation, smiling, mimicking and facial expressions. Sensory deficit can be observed affecting auditory, olfactory and visual capacities. All of these severe health problems generate situations of functional handicap. Moreover, facial appearance negatively impacts the quality of life of patients and their families. Psychological and emotional suffering and social difficulties affecting school life and professional integration are frequently reported [9, 10]. The rarity and the complexity of these diseases also cause some other difficulties, such as access to diagnosis and care [11].

Orofacial functions belong to the first stage of physiological and primary needs. They are essential to daily well-being and are prior to the needs for security, belonging, self-esteem and achievement [12]. For these reasons, it is essential that these diseases are properly and collectively managed throughout the lifetime following the evolving needs of patients. Given the variety of genotypes and treatments, this implies an integrated network of practitioners including geneticists, biologists, surgeons, paediatricians, orthodontists, dental surgeons, speech-language pathologists and psychologists. The care pathway of most of these diseases often begins with surgery. There may be reiterative surgical procedures in the early years of life and even during the teenage and young adult phase [13–15]. Parents with children suffering from rare diseases regularly express concerns about the long-term health outcomes for children who are born with an oral cleft, for example [16, 17]. Existing studies on Head, Neck and Teeth RD are often case reports and/or gene mutation reports for one disease. Very few studies have been published based on population-level data collection. There are existing epidemiological studies, based on large cohorts in Ethiopia and UK [18, 19], of the most frequent RD (when these are grouped together) such as oral cleft [18, 19]. In France there are regional data but no presently available national-level data on these diseases [20].

The RD Healthcare network in France was first organized in 2005. The Head, Neck and Teeth National Network is one of the 23 French RD healthcare networks created under the national policy on RD (http://www.orpha.net/actor/EuropaNews/2006/doc/French_National_Plan.pdf. One hundred and thirty-one expertise centres called reference centres for RD have been established in France, creating highly specialised university and non-university hospital teams for diagnosis and care. A national repository of data on patients with rare diseases, referred to centres involved in one of the rare disease networks, was established during the second French national plan. It is called BNDMR (Banque Nationale de Données Maladies Rares) (http://enlord.bndmr.fr/#homepage). Each sector includes RD reference centres and RD competence centres. Centres of expertise are called centres of reference in France, and the regional centres are called competence centres. Reference centres are intended to coordinate the definition of referential and therapeutic protocols as well as epidemiological supervision and education and research activities. At regional level, the competence centres identified by the reference centres establish the diagnoses of rare diseases, implement therapies when available, and organise patient care in relation to the designated reference centres and actors, and health and medico-social structures [21]. Reference and competence centres work with many health facilities, through diagnostic laboratories, medico-social professionals, fundamental, clinical and translational research teams, and patient associations.

Data collected from these centres are recorded in The French Rare Diseases Repository (BNDMR) with the following objectives:

1. To measure and describe the time taken to diagnose and therapeutic care. The purpose is to be able to prioritise actions to facilitate the orientation of patients in the health system when there is no specific reference centre for the concerned or suspected rare disease.
2. To improve the continuity between the actors involved in medical care, diagnostic innovation and therapeutic research.

The objective of the present study was to describe and analyse the relationship between the supply and the demand for care. The first aim was to describe the healthcare services available at regional and national levels for people suffering from these diseases. A second aim was to assess the level of access to care for these diseases through the national network. Observations at regional and national levels are presented here to better understand the patient care pathway within this specific network.

## Methods

### Study design

This was a prospective population-based study. Data were entered into the BNDMR prospectively using the CEMARA software [22]. A minimum data set (MDS) to be collected for each patient with a rare disease has been defined [23] (see Additional file 1).

This MDS was defined following a long process involving health professionals of the reference centres for RD and a ministerial working group. The MDS aims to minimize the amount of data collected by a centre while guaranteeing the quality of information collected and its exploitation. It supports communication with the information systems of hospital care. It thus constitutes a base of information common to all the uncommon diseases and all the actors involved in their care [22]. The national MDS for RD is constructed in the following way: assent/consent (regulatory), national identification of patients, personal information, family information, vital status, course of care, activity of care, history of disease, diagnosis, confirmation of the diagnosis, treatment, ante and neonatal data, research protocol, structure of care.

### Inclusion criteria

For this study, 2008–2015 data were used [22]. All patients with a head, neck or teeth RD confirmed as diagnosed by the RD network were included, with coding using the Orphanet nomenclature and/or a description of chromosomal abnormalities [24].

A “case” was defined as a patient with diagnostic certainty followed up in a RD reference centre. In some cases, tele-expertised cases may be reported and also expert opinion on medical files. Patients may be under-reported, as not all patients are referred to a centre of the network.

### The care network

#### National health network

**Fig. 1:**
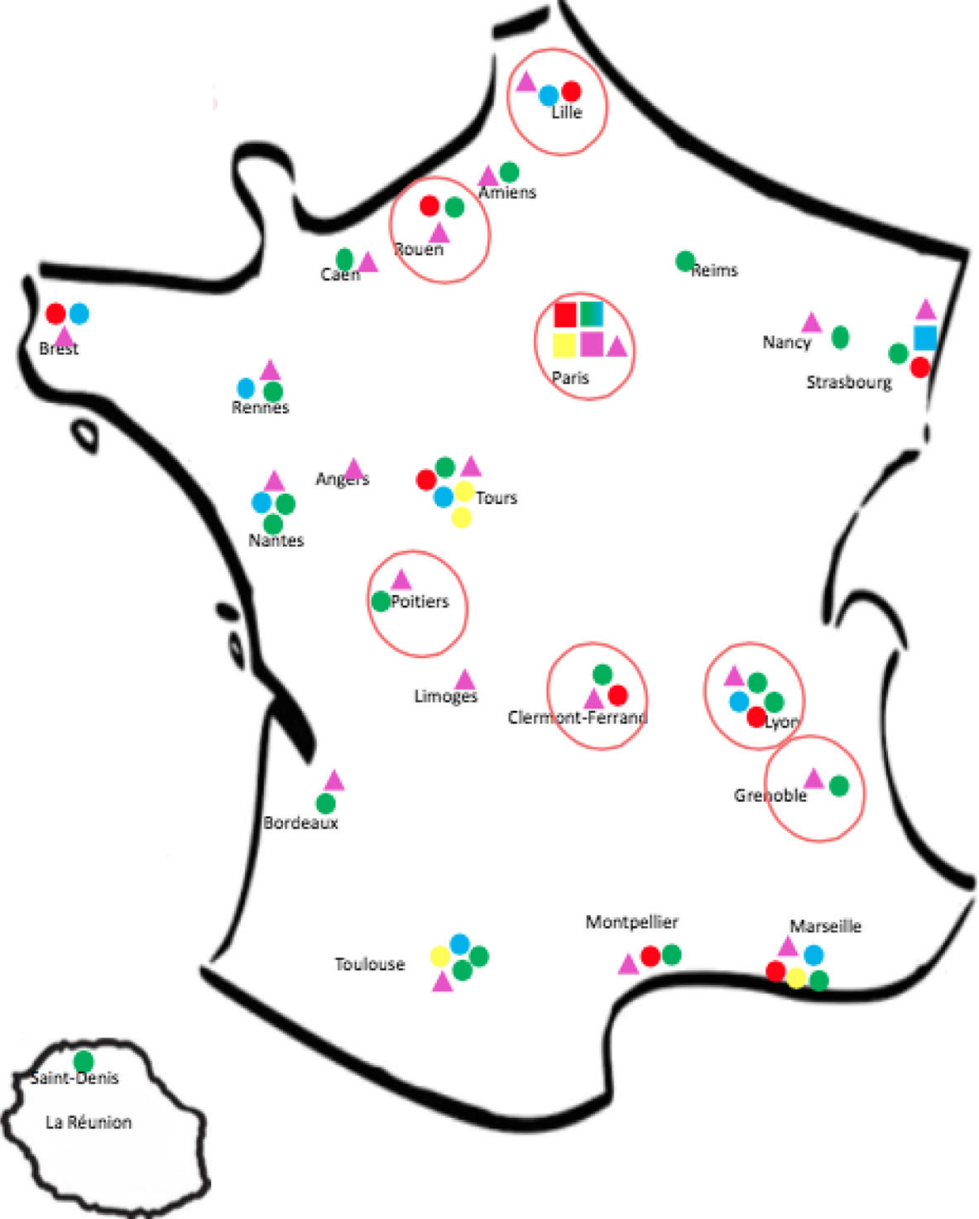
Head, Neck Teeth French Network.  CEMARA USERS.  Reference centers.  Severe ENT Malformations competence centers.  Severe Craniofacial malformations competence centers.  Cleft lip and palate competence centers.  Rare dental Malformations competence centers.  Reference centers “Pierre Robin”

National network of centres of expertise for patients with head and neck rare diseases includes six Reference Centres (Paris (4), Lille (1) and Strasbourg (1)) and 34 Competence Centres in 18 French cities. In Paris Region, four reference centres and no competence centres exist (Fig. 1). We first analysed data from all the centres. Then, to assess the balance between demand for and supply of care, we analysed data in two of the Paris reference centres, MAFACE (specialized in orofacial diseases) and MALO (specialized in othorynolaryngology). These two centres were highlighted for this analysis because they offer the most comprehensive and therefore representative data on their activity.

#### Users

In RD centres, data were coded by all the practitioners involved in care, and by non-medical staff (psychologists, speech therapists…), and were entered by medical secretaries.

#### Regulatory approval

The database was previously approved by national authorities (CCTIRS and CNIL) under authorisation number: 1187326. Patients identified in the database were informed that their data could be used for research purpose.

#### Statistical method

For this study analysis, nominative data captured were de-identified [22, 25]. An anti-duplicate device was set up automatically.

To guarantee an optimal control of the data quality, automated completeness and consistency checks were implemented. BNDMR data-managers routinely perform “queries” on themes of consistency. To verify the quality of data, the script is checked on a weekly basis by BNDMR computer scientists entirely devoted to this task. Duplicates are systematically sought and missing data in the minimal data set are verified. Each numeric variable has unauthorised values (e.g., birth date or aberrant birth weight). There are also boundaries for each numerical variable (eg a patient’s date of birth may not be later than the present day).

Data analysed were, first, population descriptions for the French regions and Paris Region (age, sex, abnormalities diagnosed), time and age at first signs, time and age at diagnosis. Next, supply of care was described (type of consultations, patient’s reference).

Two measures were performed:

- The proportion of patients in French regions was evaluated as follows: people living in the region and registered in this region during the study period (patients seen in the reference/competence centres for one of the diseases and described as living in the region) are called “patients”. The proportion was calculated as follows: P = (patient living in the region and registered in the database during the study time)/(population in the region).
- Second measure: “included cases geography”.

In order to detect possible health service provision issues, we supposed that the Paris regional data can be used as a model to project onto other regions to estimate the number of rare Head, Neck and Teeth rare diseases patients. This procedure was justified by the fact that there is no scientific proof that these diseases are, in France, related to the area of residence. Moreover, Paris is the capital of the country with many university hospitals; it is the place of choice to seek care. We standardised the Paris Region proportions onto the other French regions, adjusting for local populations. We calculated an observed proportion and an expected proportion for each region. The ratio of these proportions provides information about the numbers of patients who leave their region to find treatment in Paris Region.

The proportions of crude and standardised RD prevalence based on age, gender and national and regional localisation, have been presented with 95% confidence intervals using a Poisson model. An analysis of the distance between the place of residence and the treatment site was carried out, and the time to diagnosis for patients without confirmed diagnosis was assessed.

The statistical software used is R for Windows, version 3.3.2.

## Results

### Population description

During the period from January 2008 to December 2015, 11,342 patients in France were diagnosed with RD and entered in the database. Seventy two thousand nine hundred four of these patients were coded in the Paris Region, and 4048 outside of Paris/other French regions (Table 1).

**Table 1:** Population description

Characteristics	Patients (national except Paris Region) *n* = 4048	Patients (Paris Region) *n* = 7294
Age (years), median	7	6
(Minimum-maximum)	(0–55)	(0–55)
Sex, n =	4015	7192
Male	2160 (53.8)	3569 (49.6)
Female	1855 (46.2)	3623 (50.4)
Abnormalities diagnosed antenatally, *n*=	3290	4989
No	2148 (65.3)	3509 (70.3)
Yes	1142 (34.7)	1480 (29.7)
Other diagnoses, n	520	462

Abnormalities were diagnosed antenatally for 34.7% (*n* = 1142) of patients in other French regions and 29.7% (*n* = 1480) of patients in Paris Region (Table 1).

### Diagnosis

There were 520 clinical entities diagnosed (ORPHA code numbers) in other French regions and 462 in Paris Region. Estimated prevalence for all these diseases was 1.6 per 10,000 inhabitants (95% CI 1.0-2.5) in other French regions, and 5.1 per 10,000 inhabitants (95% CI 3.9-6.6) in Paris Region.

As shown in Table 2, the five most frequent diseases of the pathway were cleft lip and alveolus (*n* = 2059, 18.1%), cleft palate (*n* = 1799, 15.9%), vascular anomaly or angioma (*n* = 964, 8.5%), isolated Pierre Robin Syndrome (*n* = 883, 7.8%) and cleft hard palate (*n* = 688, 6.1%).

**Table 2:** Time and age at diagnosis of the 5 more frequent diseases of the network (French regions And Paris Region)

Disease	N (%)	Location	n (%)	Time at diagnosis			Age at diagnosis (months)				
				Antenatal	birth	Postnatal	Min.	1st Qu.	Median	3rd Qu.	Max.
Cleft lip and alveolus	2059 (18.1%)	French Regions	954 (16.8)	39.5	18.7	0.2	36.0	45.5	55.0	64.5	74.10
141,291		Paris Region	1105 (13.6)	27.0	3.7	0.0	0.0	0.0	0.0	0.0	0.0
Cleft palate	1799 (15.9%)	French Regions	731 (12.9)	10.5	34.3	1.5	1.0	7.5	30.0	39.0	60.0
2014		Paris Region	1068 (13.1)	15.8	8.9	0.5	1.0	1.0	4.0	18.5	120.0
Vascular anomaly or angioma	964 (8.5%)	French Regions	148 (2.6)	0.0	3.4	0.7	N/A*	N/A	N/A	N/A	N/A
68,419		Paris Region	816 (10.0)	0.0	1.8	1.7	0.0	1.0	1.0	3.5	36.0
Isolated Piere Robien Syndrome	883 (7.8%)	French Regions	318 (5.6)	7.2	56.0	1.9	2.0	2.0	2.5	32.25	120.0
718		Paris Region	565 (7.0)	5.1	35.0	1.4	0.0	1.5	2.0	13.0	48.0
Cleft hard palate	688 (6.1%)	French Regions	393 (6.9)	9.1	62.3	4.3	1.0	2.5	24.0	51.0	324.0
101,023		Paris Region	295 (3.6)	16.9	6.4	0.7	1.0	2.25	3.5	4.75	6.0

*N/A This means that there is no available data or the data is missing

Time of diagnosis was mostly antenatal for cleft lip and alveolus, with 39.5%, and 27.0% in other French regions and Paris Region respectively. Diagnosis of Isolated Pierre Robin Syndrome was at birth for 56.0% and 35.0% in the two areas. It was postnatal for 15.1% (*n* = 405) and 24.8% (*n* = 642) (Table 2).

The median age of patients at the time of diagnosis depended on the disease. For cleft lip and alveolus, median age was 55 months in the other French regions and at birth for Paris Region. For cleft palate, it was 30 months for other French regions and 4 months for Paris Region. For Isolated Pierre Robin Syndrome, it was nearly the same: 2 months and 2.5 months respectively in other French regions and Paris Region. For cleft hard palate, it was 24 months for other French regions and 3.5 months for Paris Region (Table 2).

### Supply of care

As shown in Table 3, among the different objectives of patient consultations are follow-up, global care, diagnosis, medical, emergency, prenatal diagnosis and research.

**Table 3:** Supply of care: Nature of consultation and reference of patients for other French regions and Paris Region

Characteristics	French Regions, *n* = 4048	Paris Region, *n* = 7294
Consultations, *n*=	13,445	36,039
Follow-up	7463 (55.5)	20,560 (71.8)
Global care	4164 (31.0)	9691 (33.9)
Diagnosis	849 (6.3)	3553 (12.4)
Medical act	838 (6.2)	1647 (5.7)
Emergency	39 (0.3)	459 (1.6)
Prenatal Diagnosis	82 (0.6)	73 (0.2)
Protocol	10 (0.1)	56 (0.2)
Reference centres, n	2	4
Competence centres, n	34	0
Patients referred by, *n*=		
Pediatrician	1849 (45,6)	2986 (40.9)
Other specialist	843 (20.8)	2381 (32.6)
Himself/herself or parents	323 (7.9)	798 (10.9)
Other/unknown	604 (14.9)	748 (10.3)
General practitioner	184 (4.5)	171 (2.3)
Pluridisciplinary prenatal centre	109 (2.7)	99 (1.4)
Association	62 (1.5)	31 (0.4)
Treatment centre	12 (0.3)	48 (0.7)
Geneticist	22 (0.5)	29 (0.4)
Neonatal diagnosis centre	39 (1.0)	1 (0.1)
Mother and child medical centre	1 (0.0)	27 (0.4)

Follow-up consultations represent 55.5% (*n* = 7463) of consultations in the other French regions and 71.8% (*n* = 20,560) in Paris Region (Table 3).

In terms of references, we show that patients were largely referred by paediatricians (45.6%, *n* = 1849 in other French regions and 40.9%, *n* = 2986 in Paris Region) but also by patient associations (1.5%, *n* = 62 in other French regions and 0.4%, *n* = 31 in Paris Region), or by another medical specialist (20.8%, *n* = 843 in other French regions and 32.6%, *n* = 2381 in Paris Region) (Table 3).

Some patients referred themselves or presented with a parent (7.9%, *n* = 323 in other French regions and 10.9%, *n* = 798 in Paris Region) (Table 3).

### Balance between demand for and supply of care

**Fig. 2:**
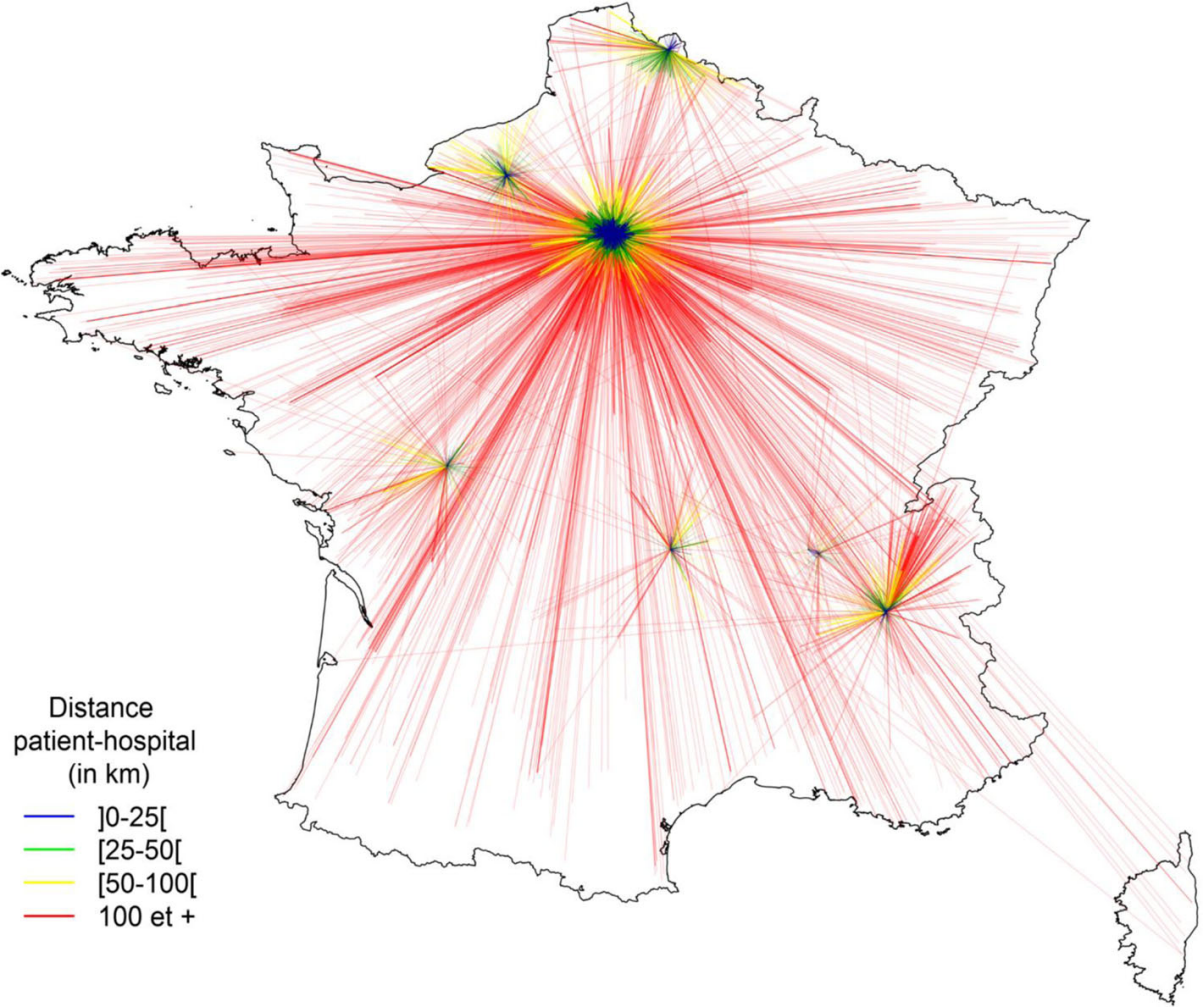
Average patient-hospital distance traveled by patients (in Km): ] 0-25 [. ] 25-50 [. ] 50-100 [.  100 and more

The care pathway of patients from French regions for treatment in Paris was analysed (Fig. 2). The figure shows the path taken by patients to seek treatment in reference and competence centres. The longer the line, the longer the journey for treatment. The thicker the line, the greater the number of patients. This figure is not quantitative, but gives an overview of the care pathways.

We analysed the number of expected and observed patients by French Region, comparing the ratios with the Paris Region (Table 4). This table shows the proportion of patients observed in each French region and the proportion expected if Paris Region was taken as a reference. The ratio of these proportions was then calculated. The closer it is to 1, the more patients have stayed in their areas for treatment. For the Haute Normandie Region (northern part of France) this ratio was 0.57. For the Midi-Pyrénées Region (southern part of France, this ratio was 0.03.

**Table 4:** Expected and observed patients by French Region, with ratio: included cases geography

Region	Population	n observed	n expected	P observed (/10,000)	P expected	Ratio
Île-de-France	11,898,502	6642	6642	5,58	5,58	1
Alsace	1,859,869	45	1038	0,24	5,58	0,04
Aquitaine	3,285,970	86	1834	0,26	5,58	0,05
Auvergne	1,354,104	236	756	1,74	5,58	0,31
Basse-Normandie	1,477,209	96	825	0,65	5,58	0,12
Bourgogne	1,641,130	201	916	1,22	5,58	0,22
Bretagne	3,237,097	155	1807	0,48	5,58	0,09
Centre	2,563,586	425	1431	1,66	5,58	0,3
Champagne-Ardenne	1,339,270	176	748	1,31	5,59	0,24
Corse	316,257	14	177	0,44	5,6	0,08
Franche-Comté	1,175,684	58	656	0,49	5,58	0,09
Guadeloupe	403,314	45	225	1,12	5,58	0,2
Guyane	239,648	18	134	0,75	5,59	0,13
Haute-Normandie	1,845,547	584	1030	3,16	5,58	0,57
Languedoc-Roussillon	2,700,266	44	1507	0,16	5,58	0,03
Limousin	738,633	35	412	0,47	5,58	0,08
Lorraine	2,349,816	59	1312	0,25	5,58	0,04
Martinique	388,364	27	217	0,7	5,59	0,12
Midi-Pyrénées	2,926,592	47	1634	0,16	5,58	0,03
Pays de la Loire	3,632,614	160	2028	0,44	5,58	0,08
Picardie	1,922,342	377	1073	1,96	5,58	0,35
Poitou-Charentes	1,783,991	319	996	1,79	5,58	0,32
Provence-Alpes-Côte d’Azur	4,935,576	96	2755	0,19	5,58	0,03
Réunion	833,944	37	466	0,44	5,59	0,08
Rhone-Alpes	6,341,160	791	3540	1,25	5,58	0,22

The activity of two reference centres, specializing in orofacial diseases and in ENT was studied (Table 5).

**Table 5:** Paris Region MAFACE and MALO reference centres (MAFACE, specializing in orofacial diseases and MALO, specializing in ENT) data: attractiveness by disease

Disease	Patients % (n)	Out-of Paris Patients % (n)
Wiedemann-Beckwith syndrome	1.0 (90)	33.3 (30)
Goldenhar syndrome	1.3 (117)	55.5 (65)
Moebius	1.0 (83)	56.6 (47)
Treacher-Collins syndrome	0.6 (56)	73.2 (41)
Hypoglossia-hypodactyly syndrome	0.1 (8)	37.5 (3)
Binder syndrome	0.3 (27)	18.5 (5)
macroglossia (except Wiedemann-Beckwith sydrom)	0.5 (44)	29.5 (13)
Arhinie and proboscis lateralis	0.1 (5)	40.0 (2)
Cleft lip with or without cleft palate/Cleft lip and alveolus/cleft palate	36.1 (3094)	32.1 (994)
BOR syndrome	0.6 (11)	45.4 (5)
CHARGE syndrome	0.6 (12)	41.7 (5)
Weissenbacher-Zweymuller syndrome	1.5 (27)	29.6 (8)
Cysts and fistulae of the face and oral cavity/Familial thyroglossal duct cyst/Congenital laryngeal cyst/Nasolacrimal duct cyst	0.5 (9)	22.2 (2)
External auditory canal aplasia/hypoplasia/anotia/microtia	23.4 (425)	42.2 (182)

We observed the proportion of patients residing outside Paris Region and coming to these centres for treatment for Wiedeman-Beckitwh syndrome, Goldenhar syndrome, Moebius syndrome, Treacher-Collins syndrome, Hypoglossia-hypodactyly syndrome), Binder syndrome, macroglossia (except Wiedemann-Beckwith sydrom, Arhinie and proboscis lateralis, Cleft lip with or without cleft palate and Cleft lip and alveolus/cleft palate.

To give more details on the medical reasons why patients residing outside the Paris Region attend the reference centres of Paris, we observed (Table 6), again by centre (MAFACE and MALO), the distribution of patients residing outside Paris Region and coming to Paris for treatment according to the objective of their consultation.

**Table 6:** By centre (MAFACE, specializing in orofacial diseases and MALO, specializing in ENT), distribution of patients residing outside Paris Region and coming to Paris for treatment per the objective of their consultation

Center	Disease	Diagnosis % (n)	Treatment % (n)	Follow-up % (n)	Medical act %(n)	Out-of Paris Global % (n)	Global CENTER (n)
MAFACE	Goldenhar	38.0 (27)	52.6 (161)	52.6 (300)	60.9 (42)	51.2 (530)	1016
	Moebius	60.9 (56)	49.5 (101)	54.4 (241)	26.7 (4)	53.3 (402)	754
	Treacher-Collins	75.9 (22)	80.3 (163)	78.2 (248)	71.6 (48)	78.1 (481)	616
	Van der Woude Syndrome	20.0 (4)	30.1 (25)	31.7 (97)	34.3 (12)	31.1 (138)	444
	Cleft lip with or without cleft palate/Cleft lip and alveolus/cleft palate	25.6 (312)	29.7 (1669)	28.5 (5297)	31.0 (514)	28.8 (7792)	27,054
MALO	BOR syndrome	66.6 (2)	66.7 (4)	0 (0)	0 (0)	46.1 (6)	13
	CHARGE Syndrome	33.3 (1)	80.0 (4)	20 (1)	0 (0)	46.1 (6)	13
	Weissenbacher-Zweymuller syndrome	0 (0)	25.0 (3)	29.4 (5)	0 (0)	27.6 (8)	29
	Cysts and fistulae of the face and oral cavity/Familial thyroglossal duct cyst/Congenital laryngeal cyst/Nasolacrimal duct cyst	66.7 (2)	0. (0)	(0) 0	0 (0)	22.2 (2)	9

For Goldenhar syndrome, for example, the proportion of patients not residing in Paris Region but treated in Paris is high (*P* = 55.5%). More patients came to have a medical procedure (*P* = 60.9) than to have a diagnosis (*P* = 38.0%). For Moebius syndrome, this proportion was reversed and more patients came for a diagnosis (*P* = 60.9) than for a medical procedure (*P* = 26.7%). This was especially true for cysts (Cysts and fistulae of the face and oral cavity/Familial thyroglossal duct cyst/Congenital laryngeal cyst/Nasolacrimal duct cyst) where patients came overwhelmingly for diagnosis (*P* = 66.7%) rather than for medical treatment (*P* = 0).

## Discussion

The epidemiological study reported here provides the first information on rare diseases of the head, neck and teeth in terms of public health in France. This is the first time that data from a national database concerning these diseases have been analysed in France.

This main results show a gap between demand for and supply of care, and reveal how patients travel from their place of residence to the reference centres in Paris with a view to recourse care and management of these very rare diseases.

According to our data, nearly 45% of all patients resided in Paris Region. This disparity between patients From Paris Region and in other French regions is probably to be explained in part by difficulties experienced by the medical teams in completing the database.

Patients were mostly diagnosed very early or at birth. This is encouraging in terms of the network’s ability to meet the patient’s need for early management, which is the best guarantee of success in the treatment of these diseases [26]. Early diagnosis does not depend on the centre (regions versus Paris). The clinical entities represented in the cohort are predominantly early-onset ones (oral clefts are becoming better diagnosed using ultrasound since this became a mandatory part of the checklist [27]).

The five most frequent diseases accounted for nearly half of all patients. These most common diseases (oral clefts and Pierre Robin Syndrome) were those treated by the two Paris centres that completed the database the most.

Our study confirmed that cleft lip and alveolus and cleft palate represented the most frequent disease type, as previously reported [28]. In the literature, some diseases (for instance cleft lip and palate) might appear at first sight not to be rare. For example, what was called an “isolated” cleft lip and or/palate could sometimes not be considered as a rare disease (occurring in one in 750 births) [20]. But the so-called “isolated clefts” in fact group together a great number of different phenotypes and aetiologies due to genetic or environmental factors and teratogenic substances, and must be studied in the same way as the syndromic forms in order to be able to count them according to their aetiology [29].

Their frequent appearance has been explained as a product of patient recruitment and because large numbers of them were coded by centres specialising in these diseases [30]. However, prevalence variations for diseases such as oral cleft may be explained by different factors. The use of the term “clefts” as if all these conditions were one phenotype may be considered a mistake. In global terms, isolated palate cleft falls within the definition of “rare” as the global prevalence is less than 1 per 2000. It also differs chronologically, anatomically, epidemiologically, developmentally, in its genetics and environmental risk factors – and in the approach to its management. Cleft Lip and/or Palate segregate separately in pedigrees and do not cross over, so they are absolutely and fundamentally different [31].

As shown in the previous results about “out-of-Paris-patients” coming to Paris for care, the finding may signify that a large number of patients were diagnosed in their regional competence and reference centres and then referred to Paris for care and/or follow up. It may therefore show that the Paris Region reference centres have a secondary health care role. It is noticeable in the results that the more specialised the centre, the more it detects and quickly diagnoses the clinical entities. Moreover, as the Paris centres also appear as centres of second resort, delays may sometimes be longer before a confirmed diagnosis is made.

In Paris, most of the medical and non-medical staff in reference centres use the database. This is why we decided to use the Paris data as a proxy to discuss the whole-of-France data (in view of the fact that the literature concerned has not shown ecological or environmental variables influencing the diseases studied) [32–34]. This may explain why the prevalence differed between Paris Region and the rest of France.

Prevalences found in the cohort for Paris Region were consistent with those of Orphanet [3], where the most frequent clinical entities were also cleft lip and alveolus, cleft palate, cleft lip and/or palate, vascular anomaly or angioma, isolated Pierre Robin Syndrome and hard palate cleft.

The ratio between proportion observed and proportion expected showed that patients living in the South of France were more apt to travel to be cared for than those living in Haute-Normandie for example.

In some areas such as South of France or Alsace, therefore, low prevalence is likely to be related to non-fulfilment of the database criteria, and may not just occur because patients are leaving their own region to be treated elsewhere. This was also true for the other regions cited. Figure 1 confirms that these regions have competence and reference centres but do not yet have full compliance with the database requirements.

In Paris, reference centres cover rare ENT malformations, Pierre Robin syndrome and congenital deglutition-suction disorders, dental manifestations of RD, craniosynostosis and craniofacial malformations, and malformations of the face and the oral cavity. Hyper-specialisation of these centres was undoubtedly an attractive feature for patients. This explains how, when diseases are very rare, such as Moebius syndrome, patients make the move to seek an expert opinion in Paris reference centres (Table 5). It was observed that the more rare the disease was, the greater the proportion of patients residing outside Paris (for example, for Treacher Collins where the proportion of patients is *P* = 0.6%, this proportion was 73.2%.) The same distribution has been described for Moebius syndrome. The proportion of patients suffering from this disease was low as recorded in the reference centre (*P* = 1.0%), but the proportion of those residing outside Paris Region is close to half of all patients. On the other hand, for oral clefts, which are very well treated in the MAFACE centre, and where the proportion of patients treated there was high, the proportion of patients residing outside Paris Region is lower than for the diseases mentioned above. “Simplicity” of cases does not necessarily imply higher prevalence. Oral clefts, for example, represent the most important pevalences, all anatomical and syndromic forms combined, nationally and regionally. The therapeutic arsenal can be very complex and sometimes needs to repeat surgeries that have failed, or to re-intervene several times before obtaining a satisfactory result.

However, other determinants of recourse or lack of care, such as inequalities faced by patients in their place of residence, should not be forgotten [35]. Measuring the supply of health care requires different indicators of the medical activity in a geographical unit and, in the case of RD, in reference centres: the establishments, their capacity and specialties, the number of doctors, general practitioners and specialists, nurses, and other paramedical professions. It is also necessary to estimate their accessibility and degree of activity. This is what our work did through this particular pathway [36–38].

### Strengths

The research project to exploit the data collected by the database aims, first of all, to obtain some epidemiological estimation of these diseases in France. Analysis of the demand for care and the supply of care, and of the balance between the two, was the primary methodological goal of the exploration of these data. In terms of the organisation of the territorial network of expert centres, the fact that many centres were not in a position to participate in the database meant that the data collected were not yet exhaustive. Indeed, the results obtained for the whole country differed significantly from the figures found in scientific literature, except for the previously discussed cleft palate results for Paris Region. It was for this reason that we made use of the results obtained in Paris Region, which were likely to be closer to epidemiological reality.

In many European countries, federative RD management has been promoted and supported by health ministries and agencies. But France is, with Spain [39], the European country where the organisation of the coverage of RD is the most institutionalised. The data collected can be used as a tool to produce descriptive indicators, but also to assess the fit between health services and needs [40, 41]. This first French national-level study may provide a methodological model for other RD data analysis. It may therefore have important potential for replication for the modelling of other RD health sectors. The reference centres, vetted by the Head, Neck and Teeth network, have received support to deploy human resources with the aim of organising the territorial rare disease network.

In terms of RD institutional organisation, France is a relatively advanced country. The Network improves access to diagnosis, treatment and the provision of high-quality healthcare to patients who have conditions requiring a particular concentration of resources or expertise. These concentrations could also be focal points for medical training and research, information dissemination and evaluation, especially for rare diseases.

In the United States, the National Organization for Rare Disorders (NORD) provides information for patients and their families, offers help to patients who cannot afford the required care, and links patients with many patient organisations that provide lists of medical experts. To find out if a particular disease has an organisation, the patient has to search in the NORD’s organisational database because there is no national institutional network especially devoted to RD [42–44]. By contrast, Italy was one of the first Member States in the European Union to regulate the field of RD [44]. In Italy, the development of a national plan or strategy for RD derived from the necessity to fulfil the EU Commission Recommendation to adopt national plans or strategies for RD by the end of 2013 [45]. In Spain, the Spanish Rare Diseases Registries Research Network is a project which aims to build a National Rare Diseases Registry based on the input of two different methods: patient outcome research registries and population-based registries [39].

### Limitations

A good network has been built in the past 8 years, but there are still some pitfalls in the analysis and also apparent issues in the patient health care circuit.

In the database, observational data were not collected for research purposes. This is why we had to deal with limitations and biases such as knowledgeable data users time-varying clinical workflows, using idiosyncratic coding practices, and showing lack of motivation to collect data and misunderstanding of purposes… If centres are not participating in the database, this implies a selection bias that tends to under-estimate the number of patients. It is for this reason that we have taken Paris, whose prevalences are close to literature ones, as a proxy. Analysis for each French Region shows the major differences that appear for many regions, where the fit between supply and demand for care seems to be unsatisfactory (Fig. 1). The ratios between observed and expected numbers calculated for the different French regions compared to Paris Region clearly shows the tendency for patients to come to Paris for their treatment.

Hospital coding for RD, using current coding and classifications systems and practices, tends to under-represent RD cases. Hospital databases are made to evaluate hospital activity. They are not intended to produce epidemiological data. Indeed, the computer systems currently present in French hospitals do not specify the rarity of clinical entities, and demonstrate the need for specialised databases [46].

To make data comprehensive and achieve exhaustiveness, it will be fruitful to cross-reference the data with additional databases such as the Medical Information Systems Program (MISP) and the National Health Insurance Fund. Patients who have not encountered the care system during the study period, or patients who have been treated for a long time, who are still living and appearing on older databases, might also be included in the registry. In this sense, the development of the BNDMR and the MDS aims to foster communication with computerised hospital care systems, using an anonymous national identifier compatible, technically and legally, with other national databases [35].

## Conclusions

Thanks to public policies and local actors, a health network covering the RD problematic from diagnosis through to research has been developed in France in the past ten years. Basic knowledge of a number of clinical entities has increased, but without appreciation of the cumulative weight of these illnesses or of patient care pathways.

At the present time, health care supply and demand are still insufficiently matched. According to the various national plans for rare diseases plans, there are reference and competence centres all over the country. Despite this, some patients are crossing the entire country to seek treatment in Paris. The rarer and more complex the disease, the more expert advice will be solicited. Hyper-specialisation of care, lack of patient information, and a deficit of resources in some centres may be elements contributing to adverse impacts on patients’ quality of life and to the heavy economic and social burdens borne by patients and their families.

Further coordination and improvement of the country-wide care network will be needed to fulfil the objective of impacting positively on the daily lives of patients. This study summarises the present situation, as a first landmark in this national–scale process, with the first confident estimations of frequencies in the French population of cleft palate, the most notable orofacial rare malformation.

## Additional file

Additional file 1: Reference and competence centres for the Head, Neck and Teeth French Rare Diseases Network. (DOCX 27 kb)

## Abbreviations

BNDMR: French rare diseases repository
CEMARA: Rare disease data bank
MDS: Minimal data set
RD: Rare disease
