# Supplementary material for: Management of rare diseases of the Head, Neck and Teeth: results of a French population-based prospective 8-year study
Source: Orphanet J Rare Dis. 2017 May 19;12:94. doi: 10.1186/s13023-017-0650-0 (PMC5437557; doi:10.1186/s13023-017-0650-0)
Supplement: Additional file 1: — Reference and competence centres for the Head, Neck and Teeth French Rare Diseases Network. (DOCX 27 kb) [file 13023_2017_650_MOESM1_ESM.docx]

| **Reference Center for Rare OroFacial Malformations (MAFACE)** Hôpital Necker-Enfants Malades/Hôpital Rothschild, Paris (Pr Marie-Paule VAZQUEZ) | | | |
| --- | --- | --- | --- |
| **Coordinator site** Hôpital Necker-Enfants Malades, Paris (Pr Marie-Paule VAZQUEZ) | | | |
| **Odontological center** Hôpital Rothschild, Paris (Pr Ariane BERDAL) | | | |
| **Reference center for Rare CRANIO-MAXILLO-FACIAL Malformations** CHRU de Lille (Pr Matthieu VINCHON) | | | |
| **Reference center for rare ENT malormations** Hôpital Necker-Enfants Malades, Paris (Pr Noël GARABEDIAN) | | | |
| **Coordinator site** Hôpital Necker-Enfants Malades, Paris (Pr Noël GARABEDIAN) | | |  |
| **Associated expertise center** Hôpital Robert Debré, Paris (Pr Thierry VAN DEN ABBEELE) | | |  |
| **Reference center for odontologic manifestations of rare diseases**  Hôpitaux Universitaires de Strasbourg (Pr Marie-Cécile MANIERE) | | | |
| **Reference center for Rare CRANIO**  **-FACIAL Malformations** Hôpital Necker-Enfants Malades, Paris (Dr Eric ARNAUD) | | | |
| **Reference centre for Pierre Robin syndromes and congenital sucking/swallowing disorders**  Hôpital Necker-Enfants Malades, Paris (Pr Véronique ABADIE) | | | |
| **Competence center for lip and palate clefs  Reference Center for Rare OroFacial Malformations (MAFACE)**- Hôpital Necker-Enfants Malades, Paris (Pr Marie-Paule VAZQUEZ/ Pr Arnaud Picard) **Reference center for Rare CRANIO-MAXILLO-FACIAL Malformations**- CHRU de Lille (Pr Matthieu VINCHON) | | | |
|  |  |  |  |
| **RÉGION** | **VILLE ET NOM DU CCMR** | **CCMR MONO-SITE /BI-SITE /TRI-SITE** | **RESPONSABLE(S) CCMR ou SITE** (pour les CCMR multi-sites) |
| ALSACE | **STRASBOURG** | **Hôpitaux Universitaires de Strasbourg** Hôpital Civil - Chirurgie B Service de Chirurgie plastique et reconstructrice, et Chirurgie maxillo-faciale et stomatologie | **Pr Catherine BRUANT RODIER** |
| AQUITAINE /  POITOU-CHARENTES | **BORDEAUX - POITIERS** (CCMR Bi-site) | **Site 1 :  CHU Hôpitaux de Bordeaux** Hôpital Pellegrin Service de chirurgie infantile | **Pr Pierre VERGNES** |
|  |  | **Site 2 :  CHU de Poitiers**  Site de la Milétrie Service de chirurgie Pédiatrique | **Dr Jiad Noel MCHEIK** |
| AUVERGNE | **CLERMONT-FERRAND** | **CHU Estaing de Clermont Ferrand** Service de chirurgie maxillo-faciale et  chirurgie plastique | **Pr Isabelle BARTHELEMY** |
| BRETAGNE | **RENNES** | **CHU de Rennes**  Hôpital Sud Anne de Bretagne  Service de Chirurgie Pédiatrique | **Pr Olivier AZZIS** |
| CENTRE | **TOURS** | **CHU TOURS**  Hôpital de TROUSSEAU  Service de Chirurgie maxillo-faciale, plastique de la face et stomatologie | **Dr Béatrice BONIN-GOGA** |
| CHAMPAGNE-ARDENNES | **REIMS** | **CHU de Reims** American Memorial Hospital Service de Chirurgie pédiatrique | **Dr Caroline FRANCOIS** |
| LANGUEDOC ROUSSILLON | **MONTPELLIER** | **CHRU de Montpellier** Hopital Lapeyronie Chirurgie orthopédique et plastique infantile | **Dr Michèle BIGORRE** |
| LA REUNION | **SAINT-DENIS** | **CHU Félix GUYON** Service de chirurgie infantile | **Dr Luke HARPER** |
| LORRAINE | **NANCY** | **CHRU de Nancy** Hôpital Central - Pavillon Krug Service de Chirurgie maxillo-faciale et plastique | **Pr Etienne SIMON** |
| MIDI-PYRÉNÉES | **TOULOUSE** (CCMR Bi-site) | **Site 1 :  CHU Toulouse**  Hôpital des enfants  Service de Pédiatrie - Chirurgie viscérale | **Pr Philippe GALINIER** |
|  |  | **Site 2 :  Clinique Medipole Garonne** | **Dr Jacques SABOYE** |
| NORMANDIE BASSE | **CAEN** | **CHU Caen** Hôpital Côte de Nacre  Service de Chirurgie maxillo-faciale,  plastique et reconstructrice,  chirurgie orale et implantologie | **Pr Hervé BENATEAU** |
| NORMANDIE HAUTE | **ROUEN** | **CHU de Rouen**  Hôpital Charles Nicolle Clinique chirurgicale infantile | **Dr Jean-Baptiste LECA** |
| PAYS DE LA LOIRE | **NANTES** (CCMR Bi-site) | **Site 1 : CHU de Nantes**  Site de l'Hôtel-Dieu Service de chirurgie Maxillo-faciale et stomatologie | **Dr Pierre CORRE** |
|  |  | **Site 2 : Clinique Jules Verne** | **Dr Jean-Claude TALMANT** |
| PACA | **MARSEILLE** | **Assistance Publique des Hôpitaux de Marseille** Hôpital de la Timone Enfants Service de Chirurgie Plastique Pédiatrique | **Dr Nathalie DEGARDIN** |
| PICARDIE | **AMIENS** | **CHU d'Amiens** Service de Stomatologie et Chirurgie Maxillo-Faciale | **Pr Bernard DEVAUCHELLE** |
| RHONE-ALPES | **GRENOBLE - LYON** (CCMR Tri-site) | **Site 1 : CHU Grenoble**  Hôpital Couple Enfant Service de chirurgie pédiatrique | **Dr Béatrice MORAND** |
|  |  | **Site 2 : Hospices Civils de Lyon** Hôpitaux Nord Hôpital Croix-Rousse Service de Chirurgie maxillo-faciale et Stomatologie | **Pr Arnaud GLEIZAL** |
|  |  | **Site 3 : Clinique du Val d'Ouest** Ecully | **Dr Isabelle JAMES** |
| **Reference center for rare ENT malormations**  (MALO) - Hôpital Necker-Enfants Malades, Paris (Pr Noel GARABEDIAN) | | | |
|  |  |  |  |
| **RÉGION** | **VILLE ET NOM DU CCMR** | **SITE DU CCMR** | **RESPONSABLE CCMR** |
| ALSACE | **STRASBOURG** | **Hôpitaux Universitaires de Strasbourg** Hôpital de Hautepierre Pôle Tête-Cou / CETD ORL et Chirurgie Cervico-faciale | **Pr Christian DEBRY** |
| AUVERGNE | **CLERMONT-FERRAND** | **CHU de Clermont-Ferrand** Hôpital Gabriel-Montpied Service ORL et Chirurgie cervico-faciale | **Pr Thierry MOM** |
| BRETAGNE | **BREST** | **CHRU Brest** Hôpital Morvan Service d'ORL Adultes et Enfants - Chirurgie de la face et du cou | **Pr Rémi MARIANOWSKI** |
| CENTRE | **TOURS** | **CHRU Hôpitaux de TOURS**  Hôpital de Clocheville Service Chirurgie pédiatrique de la tête et du cou Unité ORL - Chirurgie cervico-faciale pédiatrique | **Pr Emmanuel LESCANNE** |
| LANGUEDOC ROUSSILLON | **MONTPELLIER** | **CHRU de Montpellier** Hôpital Gui de Chauliac Département ORL, chirurgie cervico-faciale et chirurgie maxillo-faciale et stomatologie Equipe médicale : ORL et audiologie pédiatrique | **Pr Michel MONDAIN** |
| NORMANDIE HAUTE | **ROUEN** | **CHU Hôpitaux de Rouen**  Hôpital de Charles Nicolle Service ORL Chirurgie cervico-faciale | **Pr Jean-Paul MARIE** |
| NORD PAS DE CALAIS | **LILLE** | **CHRU de Lille** Hôpital Jeanne de Flandre Service d'ORL et et Chirurgie Cervico-faciale Pédiatrique | **Pr Pierre FAYOUX** |
| PACA | **MARSEILLE** | **Assistance Publique des Hôpitaux de Marseille** Hôpital de La Timone-Enfants Service d’ORL et Chirurgie Cervico-Faciale Pédiatrique | **Pr Richard NICOLLAS** |
| RHONE-ALPES | **LYON** | **Hospices Civils de Lyon** Hôpital Edouard Herriot  Service ORL, Chirurgie cervico faciale | **Pr Eric TRUY** |
| **Competence center for odontologic manifestations of rare diseases**  CRMR Rare OroFacial Malformations (MAFACE) (MAFACE) - Hôpital Necker-Enfants Malades (Chirurgie maxillo-faciale, Pr Marie-Paule VAZQUEZ/ Pr Arnaud Picard) et Hôpital Rothschild (Odontologie, Pr Ariane BERDAL), Paris CRMR Odontologic manifestations of rare diseases , Hôpitaux Universitaires de Strasbourg (Pr Marie-Cécile MANIERE) | | | |
|  | | | |
| **RÉGION** | **VILLE ET NOM DU CCMR** | **SITE DU CCMR** | **RESPONSABLE(S) CCMR ou SITE** (pour les CCMR multi-sites) |
| BRETAGNE | **RENNES - BREST** (CCMR Bi-site) | **site 1 : CHU de RENNES Centre de Soins Dentaires** Service d’odontologie et chirurgie buccale | **Pr Jean-Louis SIXOU** |
|  |  | **site 2 : CHRU de Brest** Hôpital Morvan Service d’odontologie et chirurgie buccale | **Dr Hervé FORAY** |
| CENTRE | **TOURS** | **CHRU Hôpitaux de TOURS**  Hôpital Clocheville Service de Chirurgie maxillo-faciale, plastique de la face et stomatologie | **Dr Béatrice BONIN-GOGA** |
| MIDI-PYRENÉES | **TOULOUSE** | **CHU Toulouse**  Hôpital Rangueil-Maraîchers Service d'odontologie | **Pr Frédéric VAYSSE** |
| NORD PAS DE CALAIS | **LILLE** | **CHRU de Lille** Hôpital Roger Salengro Service d’odontologie et chirurgie maxillo-faciale | **Pr Joel FERRI** |
| PACA | **MARSEILLE** | **Assistance Publique des Hôpitaux de Marseille** Hôpital de La Timone Pôle odontologie - Centre dentaire | **Pr Corinne TARDIEU** |
| PAYS DE LA LOIRE | **NANTES** | **CHU de Nantes**  Site de l'Hôtel-Dieu Odontologie Conservatrice et Pédiatrique | **Pr Brigitte ALLIOT-LICHT** |
| RHONE-ALPES | **LYON** | **Hospices Civils de Lyon** Hôpitaux Nord Service de Consultations et Traitements Dentaires | **Pr Jean-Jacques MORRIER** |
|  |  |  |  |
| **Competence centers for canio-facial malformations** CRMR Canio-facial malformations - Hôpital Necker-Enfants Malades, Paris (Dr Eric ARNAUD) CRMR Rare cranio-maxillo-facial malformations - CHRU de Lille (Pr Matthieu VINCHON) | | | |
|  |  |  |  |
| **RÉGION** | **VILLE ET NOM DU CCMR** | **SITE DU CCMR** | **RESPONSABLE CCMR** |
|  |  |  |  |
| CENTRE | **TOURS** | **site 1 : CHRU Hôpitaux de TOURS  Hôpital de Trousseau** | **Pr Boris LAURE** |
|  |  | **site 2 : CHRU Hôpitaux de TOURS  Hôpital de Clocheville** Service de Chirurgie Maxillo-Faciale |  |
| MIDI-PYRENÉES | **TOULOUSE** | **CHU Toulouse** Hôpital Pierre-Paul Riquet  Service de Neurochirurgie | **Dr Sergio BOETTO** |
| PACA | **MARSEILLE** | **Assistance Publique des Hôpitaux de Marseille** Hôpital de La Timone-Enfants Service de Neurochirurgie infantile | **Dr Grégoire PECH-GOURG** |
| RHONE-ALPES | **LYON** | **Hospices Civils de Lyon** Hôpitaux Est / Hôpital Pierre Wertheimer Neurochirurgie pédiatrique E | **Dr Carmine MOTTOLESE** |
|  |  |  |  |
| **CENTRES EXPERTS PIERRE ROBIN** CRMR for Pierre Robin syndromes and congenital sucking/swallowing disorders - Hôpital Necker-Enfants Malades, Paris (Pr Véronique ABADIE) | | | |
|  |  |  |  |
| **RÉGION** | **VILLE ET NOM DU CENTRE EXPERT** | **SITE DU CENTRE EXPERT** | **RESPONSABLE(S) CENTRE EXPERT** |
| ALSACE | **STRASBOURG** | **Hôpitaux Universitaires de Strasbourg** Hôpital de Hautepierre Service de chirurgie pédiatrique viscérale et plastique | **Dr Isabelle KAUFFMANN** |
| AQUITAINE | **BORDEAUX** | **CHU Hôpitaux de Bordeaux** Hôpital des Enfants |  |
| AUVERGNE | **CLERMONT-FERRAND** | **CHU de Clermont-Ferrand** Hôpital Estaing |  |
| BRETAGNE | **RENNES - BREST** | **CHU de Rennes** Hôpital Sud Service de médecine de l'enfant et de l'adolescent | **Dr Swellen GASTINEAU** |
|  |  | **CHRU de Brest Hôpital Morvan** Service de Néonatalogie et Réanimation pédiatrique | **Dr Mélanie BUE-CHEVALIER** |
| CENTRE | **TOURS - LIMOGES** | **CHRU Hôpitaux de TOURS** Hôpital de Clocheville Service de médecine néonatale | **Dr Annie-Laure SUC** |
|  |  | **CHU de Limoges** Hôpital Dupuytren Service d’oto-rhino-laryngologie et chirurgie cervico-faciale | **Dr Justine LERAT** |
| ILE-DE-FRANCE | **PARIS** | **Hôpital Robert Debré** Service de réanimation et surveillance continue pédiatriques | **Pr Stéphane DAUGER** |
| LANGUEDOC ROUSSILLON | **MONTPELLIER** | **CHRU de Montpellier** Hopital Lapeyronie-Arnaud de Villeneuve Chirurgie orthopédique et plastique infantile | **Pr Guillaume CAPTIER** |
| LORRAINE | **NANCY** | **CHU de Nancy** Hôpital Central - Pavillon Krug Service de Chirurgie maxillo-faciale et plastique | **Pr Etienne SIMON** |
| MIDI-PYRENÉES | **TOULOUSE** | **CHU Toulouse**  Hôpital des enfants  Service de Pédiatrie - Chirurgie viscérale | **Pr Philippe GALINIER** |
| NORD PAS DE CALAIS | **LILLE** | **CHRU de Lille** Hôpital Jeanne de Flandre Service de chirurgie ORL et cervico-faciale | **Pr Pierre FAYOUX** |
| NORMANDIE BASSE | **CAEN** | **CHU Caen** Hôpital Côte de Nacre  Service de Chirurgie maxillo-faciale, plastique et reconstructrice, chirurgie orale et implantologie | **Pr Hervé BENATEAU** |
| NORMANDIE HAUTE | **ROUEN** | **CHU Hôpitaux de Rouen** Hôpital Charles Nicolle Service de Pédiatrie Néonatale et Réanimation | **Dr Caroline LARDENNOIS** |
| PACA | **MARSEILLE** | **Assistance Publique des Hôpitaux de Marseille** Hôpital de la Timone Service de Chirurgie Plastique Pédiatrique | **Dr Nathalie DEGARDIN** |
| PAYS DE LA LOIRE | **NANTES - ANGERS** | **CHU de Nantes**  Site de l'Hôtel-Dieu Service de Chirurgie Maxillo-Faciale et Stomatologie | **Dr Pierre CORRE** |
|  |  | **CHU d'Angers** Fédération de Pédiatrie | **Dr Bertrand LEBOUCHER** |
| PICARDIE | **AMIENS** | **CHU d'Amiens** Service de Stomatologie et Chirurgie Maxillo-Faciale | **Pr Bernard DEVAUCHELLE** |
| POITOU-CHARENTES | **POITIERS** | **CHU de Poitiers**  Site de la Milétrie Service de Chirurgie Pédiatrique | **Dr Jiad Noel MCHEIK** |
| RHONE-ALPES | **LYON - GRENOBLE** | **Hospices Civils de Lyon** Hôpitaux Est Hôptal Femme Mère Enfant Service de Réanimation Pédiatrique | **Dr Catherine MAINGUY Dr Robin POUYAU** |
|  |  | **CHU Grenoble**  Hôpital Couple Enfants Service de chirurgie plastique et maxillo-faciale | **Dr Béatrice MORAND** |
